# Hemolytic and Co-Hemolytic Activity of *Trichophyton indotineae* and Related Dermatophytes Isolated from Chronic and Recalcitrant Infections

**DOI:** 10.1007/s11046-026-01062-9

**Published:** 2026-03-09

**Authors:** Oğuzhan Bingöl, Deniz Alkaya, Aylin Döğen, Murat Durdu, Macit Ilkit

**Affiliations:** 1 Division of Mycology, Department of Microbiology, Faculty of Medicine, Çukurova University, Adana, Türkiye; 2 Department of Pharmaceutical Microbiology, Faculty of Pharmacy, Mersin University, Mersin, Türkiye; 3 Department of Dermatology, Faculty of Medicine, Adana Dr. Turgut Noyan Application and Research Center, Başkent University, Adana, Türkiye

**Keywords:** Dermatophytes, Drug resistance, Hemolysin, *Trichophyton rubrum*, *Trichophyton tonsurans*, Virulence

## Abstract

Dermatophyte infections have evolved in both clinical presentation and etiological profiles, with chronic, recurrent, and widespread forms becoming increasingly prevalent and therapeutically challenging over the past decade. These infections are predominantly caused by *Trichophyton indotineae* (ITS genotype VIII), a member of the *Trichophyton mentagrophytes* complex. The virulence mechanisms of *T*. *indotineae* remain poorly understood. To elucidate potential pathogenic factors, we investigated hemolytic and co-hemolytic (CAMP-like) activities in dermatophytes associated with recalcitrant dermatophytosis. A total of 127 isolates (117 *T. indotineae*, 5 *T. rubrum*, 3 *T. tonsurans*, and 2 *Microsporum canis*) were examined using Columbia agar supplemented with 5% ovine (COA) or equine erythrocytes (CEA). Hemolysis was more consistently observed on COA (119 isolates) than on CEA (44 isolates) following 7-day incubation at 27 °C. Additional incubation at 36 °C up to 7 days was required to enhance hemolysis detection on CEA. Overall, 93.7% of isolates exhibited hemolytic activity, whereas only 3.1% demonstrated co-hemolytic activity. Among *T*. *indotineae* isolates, 97.4% were hemolytic. These findings underscore the importance of characterizing virulence traits in dermatophytes. Additional studies are needed to clarify the role of hemolysis in chronic and recalcitrant infections, with the goal of identifying novel therapeutic targets.

**Supplementary Information:**

The online version contains supplementary material available at 10.1007/s11046-026-01062-9.

## Introduction

Dermatophytes colonize the outermost layer of the epidermis, which is rich in keratinized substrates, namely skin, hair, and nails. In addition to the production of keratinases, the production of proteases, phospholipases (which hydrolyze phospholipids into fatty acids and lipophilic compounds), and the ability to adapt changes in skin pH are key virulence mechanisms during infection [1, 2]. However, other mechanisms by which dermatophytes interact with the host remain poorly characterized. Over the past decade, the incidence of chronic, recalcitrant, and widespread dermatophytosis has increased, presenting a significant clinical challenge for both dermatologists and medical mycologists. The emerging dermatophytes include *Trichophyton mentagrophytes* ITS genotype VIII (*T*. *indotineae* lineage, TmVIII), terbinafine (TRB)-resistant *T*. *rubrum*, and TRB-sensitive *T*. *mentagrophytes* ITS genotype VII (TmVII), all of which have demonstrated a global rise in prevalence [Bingöl et al., 2026, Submitted].

Hemolysins have the capacity to damage a variety of host cell types, including erythrocytes, keratinocytes, fibroblasts, and immune cells [3, 4]. Although hemolytic activity is a well-established virulence factor in bacterial pathogens, such as *Listeria ivanovii*, its role in dermatophyte infections remains unclear [5–7]. Hemolysis involves the release of hemoglobin following erythrocyte membrane disruption by hemolysins or related cytotoxic molecules. These toxins, typically classified as exotoxins, often function through pore-forming mechanisms that compromise membrane integrity and induce cell lysis [3, 4, 7, 8]. This facilitates iron acquisition from host erythrocytes, thereby supporting microbial metabolism and enhancing tissue invasion [4, 8, 9]. In addition to promoting excessive uptake of iron, hemolysins can disrupt host membrane ion balance, trigger oxidative stress, and cause cellular damage and tissue injury. Owing to these effects, hemolysins are regarded as important virulence factors in both bacterial and fungal pathogens [8, 10]. The expression of hemolytic proteins capable of lysing red blood cells has been proposed as a fungal survival strategy [11], potentially contributing to disease severity [12], whereas loss of such activity often leads to avirulence [13].

Despite the clinical burden of chronic and recalcitrant dermatophyte infections, their underlying virulence mechanisms remain poorly understood, and the basis for their aggressive clinical course warrants further investigation [7]. *Trichophyton indotineae* accounts for > 90% of chronic dermatophyte infections [Durdu et al., 2026, In revision; In preparation]. Hemolytic activity and CAMP-like co-hemolytic interactions may synergize to promote infection persistence and tissue invasion. Furthermore, the clinical features of typical dermatophyte lesions appear to differ from those caused by TmVIII strains, potentially correlating with hemolytic activity [Bingöl et al., 2026, Submitted]. These considerations prompted us to investigate the hemolytic and co-hemolytic activities of dermatophyte isolates obtained from patients with chronic and recalcitrant infections.

## Materials and Methods

### Dermatophyte Isolates

A total of 127 dermatophyte isolates were included in this study, comprising 117 *T. indotineae*, 5 *T. rubrum*, 3 *T. tonsurans*, and 2 *Microsporum canis* strains. These isolates were obtained between October 2022 and June 2025 from patients diagnosed with chronic and recalcitrant dermatophytosis (Table S1). Species identification was previously performed using either multilocus sequence typing (MLST), targeting the internal transcribed spacer region (ITS) region, the D1–D2 domain of the large subunit (LSU), partial β-tubulin (*tubb*), and translation elongation factor 1-α (*tef-1α*) [Durdu et al., 2026, In revision], or by a combination of qPCR and the DermaGenius assay, supplemented with ITS sequencing when necessary [Durdu et al., 2026, In preparation] (Table S1). The clinical source, identification method, geographic origin, and susceptibility to TRB of each isolate are given in Table S1. Prior to testing, all isolates were subcultured on Sabouraud glucose agar (SGA; Merck, Darmstadt, Germany) and incubated at 27 °C for 10 days to confirm purity and identity [5, 6]. The antifungal susceptibility profiles of the isolates were obtained from previous studies by our group using the CLSI [Durdu et al., 2026, In preparation] or EUCAST epidemiological cut-off values (ECOFFs) for TRB [Durdu et al., 2026, In revision]. TRB-susceptibility data were available for *T*. *indotineae* and *T*. *rubrum* isolates. In the case of *T*. *indotineae*, 12 isolates were categorized as wild-type (WT) phenotype and 103 as non-WT. As for *T*. *rubrum*, two isolates exhibited WT and one non-WT. In addition to *T*. *tonsurans* and *M*. *canis* isolates, two isolates of *T*. *indotineae* and *T*. *rubrum* were not available for testing in our records (Table S1).

## Hemolytic Activity

Hemolytic activity was assessed by transferring a 5 × 5 mm fragment from the edge of a 10-day-old colony onto Columbia agar supplemented with 5% ovine erythrocytes (COA) and 5% equine erythrocytes (CEA) (RTA Laboratories, Kocaeli, Türkiye). All 127 dermatophyte isolates were tested on both media in parallel. The plates were incubated at 27 °C for 7 days followed by additional incubation at 36 °C for 1–7 days. Hemolysis was evaluated macroscopically and recorded daily [5, 7, 14]. *Streptococcus pyogenes* was used as a positive control. Complete hemolysis was defined by the presence of a complete lysis of red cells in the media around the colonies. α-Hemolysis is defined as incomplete/partial lysis of red blood cells. This causes the formation of a green or brown discoloration around the colony in the medium. There was no discernible change in the color of the medium constituting ɣ-hemolysis.

## Qualitative CAMP Test

The qualitative CAMP test was performed using COA and CEA media (RTA Laboratories) with the same panel of dermatophyte strains. Punctiform inoculation was conducted using a sterile needle. After 3–7 days of incubation at 27 °C, a sterile loop was used to streak β-hemolytic *Staphylococcus aureus* ATCC 25923, α-hemolytic *Streptococcus pneumoniae* ATCC 6303, and non-hemolytic *Enterococcus faecalis* ATCC 29212 across the plate, maintaining a 10 mm distance from the dermatophyte colony margin. Plates were further incubated at 27 °C and examined daily for up to 7 days. A positive CAMP-like reaction was defined by the appearance of a sharp, arrowhead-shaped zone of hemolysis at the interface of the dermatophyte and bacterial strains [6, 7].

## Statistical Analysis

The chi-square test was applied to statistically compare the hemolytic activities of the dermatophyte species. SPSS software (version 23, IBM, Armonk, NY, USA) was used for the statistical evaluation of the study data.

## Results

**Fig. 1:**
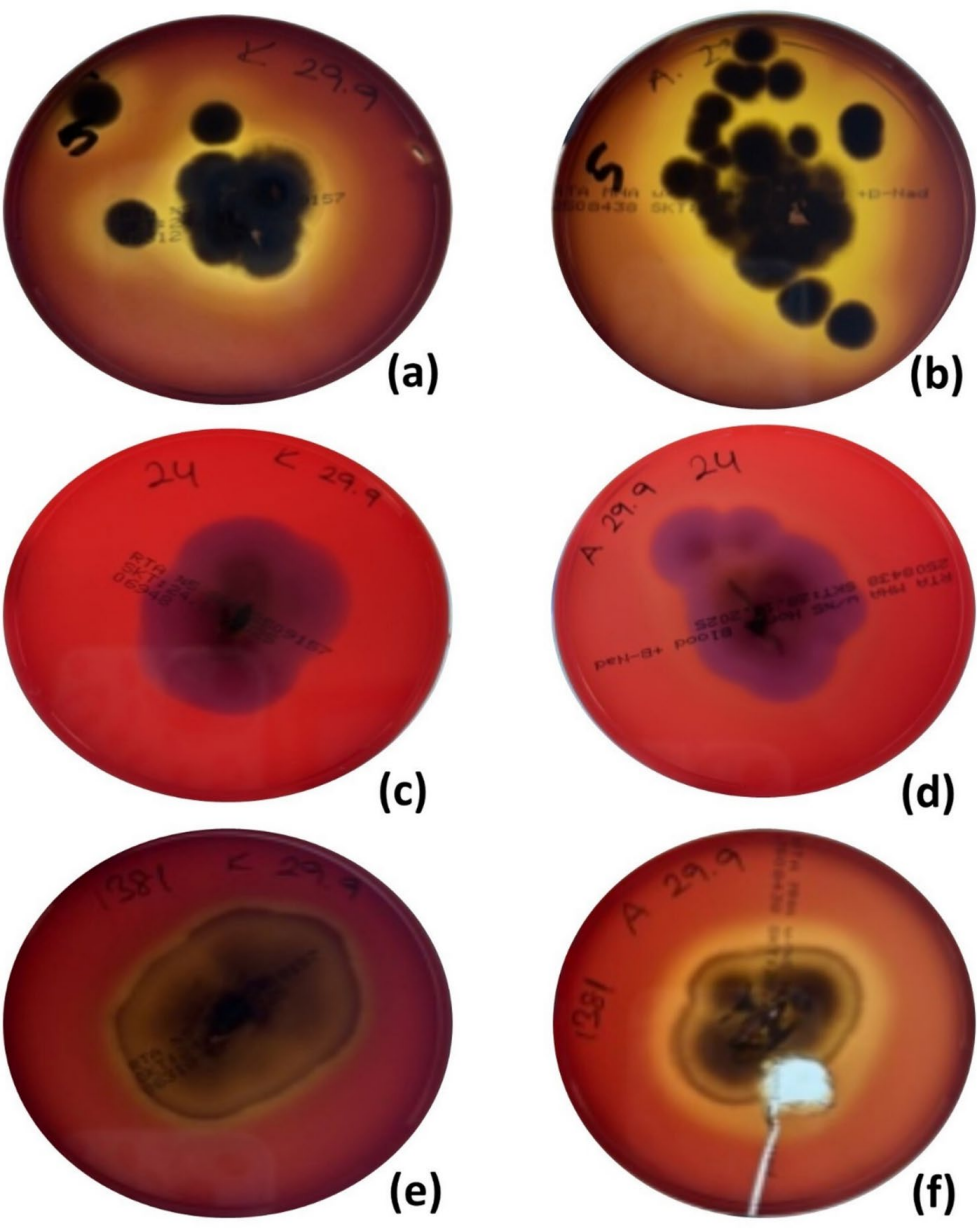
Hemolytic activity of *Trichophyton* isolates on different erythrocyte-supplemented media. All cultures were carried out for a period of 7 days at 27 °C, *Trichophyton indotineae* MI 19971 grown on (**a**) Columbia agar with 5% ovine erythrocytes (COA) and (**b**) Columbia agar with 5% equine erythrocytes (CEA); *T*. *rubrum* MI 19972 grown on (**c**) COA and (**d**) CEA; *Trichophyton tonsurans* MI 19973 grown on (**e**) COA and (**f**) CEA

Hemolytic activity varied depending on the culture medium and incubation conditions. Hemolysis was more readily detected on COA than on CEA, with 119 versus 44 isolates exhibiting hemolytic zones, respectively, after incubation at 27 °C for 7 days (Table 1; Fig. 1). In total, 119 of the 127 dermatophyte isolates (93.7%) exhibited β-hemolysis when cultured on COA (27 °C for 7 days), and 8 of the isolates (6.3%) were classified as ɣ-hemolytic. Compared with other dermatophyte species,* T*. *indotineae* isolates presented a significantly greater rate of β-hemolytic activity at 27 °C (χ^2^ = 27.54, *p* < 0.001). On CEA, hemolytic activity was not apparent under these initial conditions and required additional incubation at 36 °C for 1–7 days, during which 121 isolates developed β-hemolytic zones. α-Hemolysis was observed in 6 of 127 isolates after prolonged incubation. Although all *M*. *canis* isolates exhibited hemolytic activity, only limited activity was found in the case of *T*. *tonsurans* and* T*. *rubrum*.

**Table 1:** Hemolytic and CAMP-like activities of dermatophyte isolates recovered from recalcitrant infections

Species	Hemolytic activity at 27 °C/36 °C						CAMP-like activity	
	β-hemolysis		α-hemolysis		ɣ-hemolysis			
	COA	CEA	COA	CEA	COA	CEA	COA	CEA
*T*. *indotineae* (*n* = 117)	114/114	40/113	0/2	0/2	3/1	77/2	3	1
*T*.* rubrum* (*n* = 5)	3/3	2/4	0/2	0/0	2/0	3/1	0	0
*T*. *tonsurans* (*n* = 3)	0/2	1/3	0/1	0/0	3/0	2/0	1	0
*M*. *canis* (*n* = 2)	2/1	1/1	0/1	0/1	0/0	1/0	0	0
Total (*n* = 127)	119/120	44/121	0/6	0/3	8/1	83/3	4	1

COA, Columbia agar with 5% ovine erythrocytes; CEA, Columbia agar with 5% equine erythrocytes

Values are shown as counts of isolates with hemolytic activity observed at 27 °C for 7 days (first value) and following additional incubation at 36 °C for 1–7 days (second value)

**Fig. 2:**
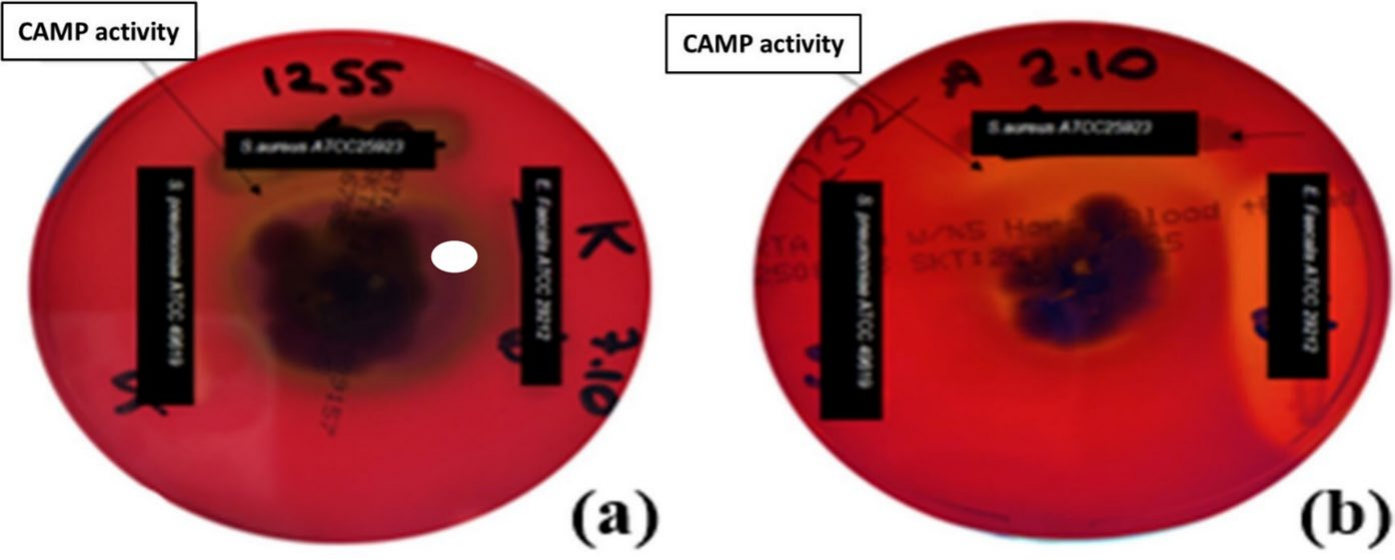
CAMP-like activity of *T*. *indotineae* MI 19974 and MI 19975 grown on (**a**) Columbia agar with 5% ovine erythrocytes (COA) and (**b**) Columbia agar with 5% equine erythrocytes (CEA). Incubation over 7 days at 27 °C

In none of the 127 isolates (whether in COA or CEA cultures) was the bizonal lytic effect apparent. Furthermore, CAMP-like co-hemolytic activity was detected in only 4 of 127 isolates (3.1%) on COA, and these isolates were strains of *T*. *indotineae* (*n* = 3) and *T. tonsurans* (*n* = 1) (Table 1; Fig. 2). Overall, COA, as a growth medium, was superior to CEA in terms of producing both hemolytic and co-hemolytic activity. CAMP-like activity was rarely observed and did not allow for robust statistical comparison due to the limited number of positive isolates. Furthermore, the limited number of TRB-sensitive isolates precluded a reliable statistical comparison with hemolytic activity.

## Discussion

In a previous study, the hemolytic activity of 76 dermatophyte isolates from patients with acute (*n* = 30) and chronic (*n* = 46) infections was evaluated, with the result that hemolysis was evident in 21.1% of the isolates, with a slightly higher prevalence in those from chronic cases (21.7% vs. 20%) [15]. However, neither the culture medium nor the erythrocyte source were specified. At the time (1994), species identification relied solely on morphological criteria, clinical presentations were relatively uniform, and *T*. *indotineae* had not yet been identified [15]. Three decades later, the global dermatophytosis landscape has shifted, marked by a surge in chronic, recurrent, and treatment-resistant infections that present with distinct clinical characteristics [16; Bingöl et al., 2026, Submitted; Durdu et al., 2026, In revision; In preparation]. In light of these developments, our primary objective was to investigate hemolytic and co-hemolytic activities in dermatophyte isolates from chronic and recalcitrant infections.

We examined 127 dermatophyte isolates from our laboratory collection (growth on both COA and CEA media) and adopted an optimized protocol for detecting hemolysis and CAMP-like activity [7]. The majority of isolates (93.7%) exhibited hemolytic activity, whereas co-hemolytic (CAMP-like) activity was detected in only 3.1% of the isolates. Hemolysis was more prevalent in those isolates cultured on COA; in the case of CEA isolates, the activity was much reduced after 7 days. These findings support the potential role of hemolysins in mediating host–pathogen interactions in dermatophytosis. However, the association between hemolytic phenotype and clinical severity or treatment outcome remains unclear. Comparative analysis with TRB-sensitive isolates may offer further insight. As in bacterial infections [3, 4], fungal hemolysins are believed to damage not only erythrocytes but also keratinocytes and fibroblasts. Notably, post-inflammatory hyperpigmentation frequently observed in *T*. *indotineae* infections may be linked to hemolytic activity, a hypothesis that warrants further investigation [16; Durdu et al., 2026, In revision; In preparation].

Disseminated fungal hemolytic infection may be a biomarker for the development of fungal hemolytic activity; however, practical methods for detecting this activity have yet to be developed [8]. The presence of certain bacteria on the skin is necessary for fungal co-hemolytic activity to occur; however, this is not the case with hemolytic activity [10] the presence of a fungus with hemolytic activity alone is sufficient. We hypothesize that positive hemolytic activity can explain the behavior of *T*. *indotineae* strains, which (regardless of topical or systemic antifungal treatments) tend to spread throughout the body rather than being limited to an anatomical area.

Species-specific erythrocyte susceptibility to a hemolysin derived from *T*. *mentagrophytes* has been reported: human erythrocytes are the most resistant, followed by those of dogs, swine, cattle, sheep, and horses [11]. This observation suggests a degree of host cell specificity, as seen with other hemolysins. The clinical relevance of hemolytic activity remains controversial, with conflicting results even among studies involving the same species, infection sites, ecological niches, and experimental protocols [5–11, 14, 15, 17, 18]. In our previous work, we found that hemolysis was best detected on CA supplemented with ovine, equine, or bovine erythrocytes [7].

Aneke et al. [17] demonstrated that 92 of 100 *M. canis* isolates produced enzymes (phospholipase, catalase, hemolysin, and lipase) regardless of their origin (human or animal) or associated clinical signs, suggesting that these enzymes are constitutive components of the enzymatic arsenal of the fungus. Hemolytic activity was observed in 92% of the isolates, reinforcing the view that this function may support fungal survival during infection [17]. Conversely, Aktaş et al. [9] did not detect hemolysis in *M*. *canis*, *E*. *floccosum*, or *T*. *violaceum* isolates. *Trichophyton rubrum*, a globally distributed species, has also shown variable hemolytic profiles. Solgun et al. [14] reported β-, α-, and ɣ-hemolysis in 26.6%, 63.3%, and 10.1% of *T*. *rubrum* isolates (*n* = 79), respectively, whereas the corresponding proportion of isolates in another study were 20.9%, 48.9%, and 30.2% of isolates (*n* = 43) [9]. In the case of *T*. *mentagrophytes* (*n* = 7), Aktaş et al. [9] detected β-hemolysis in four isolates and α-hemolysis in three. Similarly, *T*. *tonsurans* (*n* = 5) isolates displayed α- and ɣ-hemolysis in three and two isolates, respectively [9]. Bizonal lytic effect and complete and incomplete hemolytic effect were described previously in strains of *T*. *rubrum* [5, 7] and *T*. *equinum* [5], indicating the selection of two distinct cytolytic factors. In the present study, we did not observed bizonal lytic effect in isolates cultured in either COA or CEA.

CAMP-like interactions, which may contribute to dermatophyte pathogenesis, have been previously documented [6, 7, 10]. Bacterial-fungal interactions are known to compromise the skin barrier in co-infections, such as erysipelas/cellulitis with tinea pedis [1] and kerion celsi [19]. Such synergism may influence disease severity and recurrence. However, the exact membrane-damaging factors secreted by dermatophytes remain poorly defined [6]. In our study, CAMP-like activity was limited to four isolates on COA, suggesting that this mechanism plays a minor role in recalcitrant infections. This co-hemolytic phenomenon has been associated with membrane sphingomyelin content: 51% in ovine and bovine erythrocytes compared to only 13.5% in equine erythrocytes [20]. High concentrations of sphingolipids are also found in the epidermis, where they play critical roles in maintaining the skin barrier [21]. The present study has several limitations. First, TmVII, which is clinically distinct from conventional dermatophyte strains, was not represented in our isolate collection [Bingöl et al., 2026, Submitted]. Second, CA supplemented with 5% bovine erythrocytes, optimal for CAMP-like activity detection [7], was not available during the study period. Lastly, isolates from acute infections were not included for comparison.

Given the increasing incidence of chronic and resistant dermatophytosis, it is both timely and necessary to investigate the underlying virulence and pathogenic mechanisms. Our findings provide preliminary insights into the potential roles of hemolysins and related factors in infection persistence and host tissue damage. These data may inform future studies aimed at: (i) developing effective infection control strategies and (ii) countering the spread of *T*. *indotineae*, a globally emerging and drug-resistant dermatophyte. By profiling hemolytic activity in a large collection of dermatophyte isolates, we aim to contribute to the identification of novel antifungal targets. Despite the urgent need for new antifungal therapies, no new class of systemic antifungal drugs for skin diseases has been introduced in the past two decades.

## Supplementary Information

Below is the link to the electronic supplementary material.

Supplementary file1 (XLSX 28 kb)

## Data Availability

Data sets are available upon request to OB, AD, and MI.
